# Supplementary material for: Heat adaptation of phage T7 under an extended genetic code
Source: Virus Evol. 2021 Dec 1;7(2):veab100. doi: 10.1093/ve/veab100 (PMC8923235; doi:10.1093/ve/veab100)
Supplement: veab100_Supp [file veab100_supp.zip › Sequencing_Analysis.zip!/Sequencing_Analysis/S1/evidence/JC_SIDE_2_58.html]

BRESEQ :: Evidence


| New junction evidence | | | | | | | | | | | |
| --- | --- | --- | --- | --- | --- | --- | --- | --- | --- | --- | --- |
|  | | seq id | position | reads (cov) | reads (cov) | score | skew | freq | annotation | gene | product |
| \* | ? | NC\_001604.1 | = 16508 | 1353 (0.320) | 2351 (0.570) | 369/586 | 0.2 | 62.6% | intergenic (–/–) | *–/–* | –/– |
|  | ? | NC\_001604.1 | 16970 = | 1483 (0.360) |  |  |  |  | intergenic (–/–) | *–/–* | –/– |

Only 100 of 3891 total aligned reads displayed.  

`ATGCCAAGCAGGTTAGGCTAGGTGGATGCGGTAGGTCCGAATATAAGGATGGAGACTTTGACATTCTTGCGGTTGTGGTTGACGAAGATGTGCTTATTTTCACATGGGACGAAGTAAAAGGTAAGACATCCATGTGTGTCGGCAAGAGAAACAAAGGCATAAAACTATAGGAGAAATTATTATGGCTATGACAAAGAAATTTAAAGTGTCCTTCGACGTTACCGCAAAGATGTCGTCTGACGTTCAGGCAATCTTAGAGAAAGATATGCTGCATCTATGTAAGCAGGTCGGCTCAGGTGCGATTGTCCCCAATGGTAAACAGAAGGAAATGATTGTCCAGTTCCTGACACACGGTATGGAAGGATTGATGACATTCGTAGTACGTACATCATTTCGTGAGGCCATTAAGGACATGCACGAAGAGTATGCAGATAAGGACTCTTTCAAACAATCTCCTGCAACAGTACGGGAGGTGTTCTGA‑TGTCTGACTACCTGAAAGTGCTGCAAGCAATCAAAAGTTGCCCTAAGACTTTCCAGTCCAACTATGTACGGAACAATGCGAGCCTCGTAGCGGAGGCCGCTTCCCGTGGTCACATCTCG  >  NC_001604.1/16670‑17269`  

                                                                                                                                                                                                                                                                                                            `|`                                                                                                                                                                                                                                                                                                              
     `tctgcaaactgtggattatcaagaccgaagagatgctcgtagagaaaggcttgaagcatggctgggatggggactttgcgtacatggcatgggtacatgatgaaatccaagtaggcttccgtaccgaagagattgctcaggtggtcattgagaccgcacaagaagcgatgcgctgggttggagaccactggaacttccggtgtcttctggataccgaaggtaagatgggtcctaattgggcgatttgccactgatacaggaggctactcatgaacgaaagacacttaacaggtgcGATTGt                                                                                                                                                                                                                                                                                                         >  1:43520‑M2/296‑301 (MQ=255)`  
             `ctgtggattatcaagaccgaagagatgctcgtagagaaaggcttgaagcatggctgggatggggactttgcgtacatggcatgggtacatgatgaaatccaagtaggctgccgtaccgaagagattgctcaggtggtcattgagaccgcacaagaagcgatgcgctgggttggagacaactggaacttccggtgtcttctggataccgaaggtaagatgggtcctaattgggcgattttccactgatacaggaggctactcatgaacgaaagacacttaacaggtgcGATTGTCCCCAatc                                                                                                                                                                                                                                                                                                 >  1:473569‑M2/288‑300 (MQ=255)`  
              `ggggtAGGTGGATGCGGTAGGTCCGAATATAAGGATGGAGACTTTGACATTCTTGCGGTTGTGGTTGACGAAGATGTGCTTATTTTCACATGGGACGAAGTAAAAGGTAAGACATCCATGTGTGTCGGCAAGATAAACAAAGGCATAAAACTATAGGAGAAATTATTATGGCTATGACAAAGAAATTTAAAGTGTCCTTCGACGTTACCGGAAAGATGTCGTCTGACGTTCAGGCAATCTTAGAGAAAGATATGCTGCATCTATGTAAGCAGGTCGGCTCAGGTGCGATTGTCCCCAATgg                                                                                                                                                                                                                                                                                                <  1:262266/297‑1 (MQ=255)`  
              `gggctagggggtagggtaaagTCCGAATAGAAGGATGGAGACTTTGACATGCTTGCGGTTGTGGTTGACGAAGATGTGCTTATTTTCACATGGTACGAATTAAAAGGTAAGACATCCATTGGTGTCGGCAAGAGAAACAAATGTATAAAACTATAGGAGAAATTATTATGGCTATGAAAAAGAAATTTAAAGTGTCCTTCGACGTTACCGCAAAGATGTCGTCTGACGTTCAGGCAATCTTAGAGAAAGATATGCTGCATCTATGTAAGCAGGTCGGCTCAGGTGCGATTGTCCCCAATgg                                                                                                                                                                                                                                                                                                <  1:123201/281‑1 (MQ=255)`  
              `gggCTAGGTGGATGCGGTAGGTCCGAATATAAGGATGGAGACTTTGACATTCTTGCGGTTGTGGTTGACGAAGATGTGCTTATTTTCACATGGGACGAAGTAAAAGGTAAGACATCCATGTGTGTCGGCAAGAGAAACAAAGGCATAAAACTATAGGAGAAATTATTATGGCTATGACAAAGAAATTTAAAGTGTCCTTCGACGTTACCGCAAAGATGTCGTCTGACGTTCAGGCAATCTTAGAGAAAGATATGCTGCATCTATGTAAGCAGGTCGGCTCAGGTGCGATTGTCCCCAATgg                                                                                                                                                                                                                                                                                                <  1:510003/300‑1 (MQ=255)`  
              `gacagaGGGGGATGCGGTAGGTCCGAATATAAGGATAGAGACTTTGACATACTGGCGGTGGTGGTTGACGAAGAAGTGATTATTTTCACATGGGACGAAGTAAAAGGTAAGACATCCATGGGTGTCTGCAAGAGAAACAAAGGCATAAAACTATAGGAGAAATTATTATGGCTATGACAAAGAAATTTAAAGTGTCCTTCGACGTTACCGCAAAGATGTCGTCTGACGTTCAGGCAATCTTAGAGAAAGATATGCTGCATCTATGTAAGCAGGTCGGCTCAGGTGCGATTGTCCCCAATgg                                                                                                                                                                                                                                                                                                <  1:525388/296‑1 (MQ=255)`  
              `aGGCTAGGTGGATGCGGTAGGTCCGAATATAAGGATGGAGACTTTGACATTCTTGCGGTTGTGGTTGACGATTTTTTTCTTATTTTCTCTTTGGACGAATTCTCCTGTAAGACTTCTTTTTGTGTCGTCTTCTTAAACAAATTCTTATAACTATACGTCTCTTTATTATGTCTATGACATATATATTTTTAGTGTCCTTCTACGTTACCGCAAATATGTCGTCTGCCTTTTTGGCAATCTTAGAGATCGTTCTGCTGCATCTTTGTCAGCCGTTTGGCTCAGGTTCGATTGTcccttttgt                                                                                                                                                                                                                                                                                                >  1:46/1‑295 (MQ=255)`  
               `ggCTAGGTGGATGCGGTAGGTCCGAATATAAGGATGGAGACTTTGACATTCTTGCGGTTGTGGTTGACGAAGATGTGCTTATTTTCACATGGGACGAAGTAAAAGGTAAGACATCCATGTGTGTCGGCAAGAGAAACAAAGGCATAAAACTATAGGAGAAATTATTATGGCTATGACAAAGAAATTTAAAGTGTCCTTCGACGTTACCGCAAAGATGTCGTCTGACGTTCAGGAAATCTTAGAGAAAGATATGCTGCATCTATGTAAGCAGGTCGGCTCAGGTGCGATTGTccccctgtct                                                                                                                                                                                                                                                                                               >  1:588298/1‑295 (MQ=255)`  
                 `ggattatcaagaccgaagagatgctcgtagagaaaggcttgaagcatggctgggatggggactttgcgtacatggcatgggtacatgatgaaatccaagtaggctgccgtaccgaagagattgctcaggtggtcattgagaccgcacaagaagcgatgcgctgggttggagaccactggaacttccggtgtcttctggataccgaaggtaagatgggtcctaattgggcgatttgccactgatacaggaggctactcatgaacgaaagacacttaacaggtgcGATTGTCCCCAATGGTaa                                                                                                                                                                                                                                                                                             >  1:530939‑M2/284‑301 (MQ=255)`  
                   `aGGTGGATGCGGTAGGTCCGAATATAAGGATGGAGACTTTGACATGCTTGCGGTTGTGGTTGACGAAGATGTGCTTATTTTCACATGGGACGAAGTAAAAGGTAAGACATCCATGTGTGTCGGCAAGAGAAACAAAGGCATAAAACTATAGGAGAAATTATTATGGCTATGACAAAGAAATTTAAAGTGTCCTTCGACGTTACCGCAAAGATGTCGTCTGACGTTCAGGCAATCTTAGAGAAAGATATGCTGCATCTATGTAAGCAGGTCGGCTCAGGTGCGATTGTCCCCAATGGTAAAc                                                                                                                                                                                                                                                                                           <  1:135318/301‑1 (MQ=255)`  
                   `aGGTGGATGCGGTAGGTCCGAATATAAGGATGGAGACTTGGACAGTCTTGCGGTTGTGGTTGACGAAGATGTGCTTATTTTCACATGGGACGAAGTAAAAGGTAAGACATCCATGTGTGTCGGCAAGAGAAACAAAGGCATAAAACTATAGGAGAAATTATTATGGCTATGACAAAGAAATTTAAAGTGTCCTTCGACGTTACCGCAAAGATGTCGTCTGACGTTCAGGCAATCTTAGAGAAAGATATGCTGCATCTATGTAAGCAGGTCGGCTCAGGTGCGATTGTCCCCAATGGTAAAc                                                                                                                                                                                                                                                                                           <  1:582154/301‑1 (MQ=255)`  
                    `aagagacaaccgaagagatgctcgtagagaaaggcttgaagcatggctgggatggggactttgcgtacatggcatgggtacatgatgaaatccaagtaggctgccgtaccgaagagattgctcaggtggtcattgagactgcacaagaagcgatgcgctgggttggagaccactggaacttccggtgtcttctggataccgaaggtaagatgggtcctaattgggcgatttgccactgatacaggaggctactcatgaacgaaagacacttaacaggtgcGATTGTCCCCAATGGTAAACa                                                                                                                                                                                                                                                                                          <  1:415983‑M2/21‑1 (MQ=255)`  
                     `tatcaagaccgaagagatgctcgtagagaaaggcttgaagcatggctgggatggggactttgcgtacatggcatgggtacatgatgaaatccaagtaggctgccgtaccgaagagattgctcaggtggtcattgagaccgcacaagaagcgatgcgctgggttggagaccactggaacttccggtgtcttctggataccgaaggtaagatgggtcctaattgggcgatttgccactgatacaggaggctactcatgaacgaaagacacttaacaggtgcGATTGTCCCCAATGGTAAACAg                                                                                                                                                                                                                                                                                         >  1:374425‑M2/280‑301 (MQ=255)`  
                     `tatcaagaccgaagagatgctcgtagagaaaggcttgaagcatggctgggatggggactttgcgtacatggcatgggtacatgatgaaatccaagtaggctgccgtaccgaagagattgctcaggtggtcattgagaccgcacaagaagcgatgcgctgggttggagaccactggaacttccggtgtcttctggataccgaaggtaagatgggtcctaattgggcgatttgccactgatacaggaggctactcatgaacgaaagacaattaacaggtgcGATTGTCCCCAATGGTAaacca                                                                                                                                                                                                                                                                                         >  1:314769‑M2/280‑299 (MQ=255)`  
                      `atcaagaccgaagagatgctcgtagagaaaggcttgaagcatggctgggatggggactttgcgtacatggcatgggtacatgatgaaatccaagtaggctgccgtaccgaagagattgctcaggtggtcattgagaccgcacaagaagcgatgcgctgggttggagaccactggaacttccggtgtctcctggataccgaaggtaagatgggtcctaattgggcgatttgccactgatacaggaggctactcatgaacgaaagacacttaacaggtgcGATTGTCCCCAATGGTAAAAAGa                                                                                                                                                                                                                                                                                        >  1:288386‑M2/279‑301 (MQ=255)`  
                       `ggATGCGGTAGGTCCGAATATAAGGATGGAGACTTTGACATTCTTGCGGTTGTGGTTGACGAAGATGTGCTTATTTTCACATGGGACGAAGTAAAAGGTAAGACATCCATGTGTGTCGGCAAGAGAAACAAAGGCATAAAACTATAGGAGAAATTATTATGGCTATGACAAAGAAATTTAAAGTGTCCTTCGACGTTACCGCAAAGATGTCGTCTGACGTTCAGGCAATCTTAGAGAAAGATATGCTGCATCTATGTAAGCAGGTCGGCTCAGGTGCGATTGTCCCCAATTGTAAACAGaa                                                                                                                                                                                                                                                                                       >  1:177235/1‑301 (MQ=255)`  
                          `agaccgaagagatgctcgtagagaaaggcttgaagcatggctgggatggggactttgcgtacatggcatgggtacatgatgaaatccaagtaggctgccgtaccgaagagattgctcaggtggtcattgagaccgcacaagaagagatgcgctgggttggagaccactggaacttccggtgtcttctggataccgaaggtaagatgggtcctaattgggcgatgtgccactgatacaggaggctactcatgaacgaaagacacttaacaggtgcGATTGTCCCCAATGGTAAACAGAAGGa                                                                                                                                                                                                                                                                                    >  1:205852‑M2/275‑301 (MQ=255)`  
                           `gaccgaagagatgctcgtagagaaaggcttgaagcatggctgggatggggactttgcgtacatggcatgggtacatgatgaaatccaagtaggctgccgtaccgaagagattgctcaggtggtcattgagaccgcacaagaagcgatgcgctgggttggagaccactggaacttccggtgtcttctggataccgaaggtaagatgggtcctaattgggcgatttgccactgatacaggaggctactcatgaacgaaagacacttaacaggtgcGATTGTCCCCAATGGTAAACAGaaggct                                                                                                                                                                                                                                                                                   >  1:171347‑M2/274‑299 (MQ=255)`  
                                       `aaTATAAGGATGGAGACTTTGACATTCTTGCGGTTGTGGTTGACGAAGATGTGCTTATTTTCACATGGGACGAAGTAAAAGGTAAGACATCCATGTGTGTCGGCAAGAGAAACAAAGGCATAAAACTATAGGAGAAATTATTATGGCTATGACAAAGAAATTTAAAGTGTCCTTCGACGTTACCGAAAAGATGTCGTCTGACGTTCAGGCAATCTTAGAGAAATATATGCTGCATCTATGTAAGCCGGTCGTATCAGGTGCGATTGTCCCCAATGGTAAACAGAAGGAAATGATTGTCCAg                                                                                                                                                                                                                                                                       >  1:320789/1‑301 (MQ=255)`  
                                        `ctcgtagagaaaggcttgaagcatggctgggatggggactttgcgtacatggcatgggtacatgatgaaatccaagtaggctgccgtaccgaagagattgctcaggtggtcattgggaccgcacaagaagcgatgcgctgggttggagaccactggaacttccggtgtcttctggataccgaaggtaagatgggtcctaattgggcgatttgccactgatacaggaggctactcatgaacgaaagacacttaacaggtgcGATTGTCCCCAATGGTAAACAG‑ACGCAATGATTGTACAGtt                                                                                                                                                                                                                                                                     >  1:295840‑M2/261‑301 (MQ=255)`  
                                          `ataAGGATGGAGACTTTGACATTCTTGCGGTTGTGGTTGACGAAGATGTGCTTATTTTCACATGGGACGAAGTAAAAGGTAAGACATCCATGTGTGTCGGCAAGAGAAACAAAGGCATAAAACTATAGGAGAAATTATTATGGCTATGACAAAGAAATTTAAAGTGTCCTTCGACGTTACCGCAAAGATGTCGTCTGACGTTCAGGCAATCTTAGAGAAAGATATGCTGCATCTATGTAAGCAGGTCGGCTCAGGTGCGATTGTCCCCAATGGTAAACAGAAGGAAATGATTGTCCAGTTc                                                                                                                                                                                                                                                                    <  1:537357/301‑1 (MQ=255)`  
                                             `agagaaaggcttgaagcatggcttggatggggactttgcggacatggcatgggtacatgatgaaatccaagtaggctgccgtaccgaagagattgctcaggtggtcattgagaccgcacaagaagcgatgcgctgtgttggataccactagaacttccggtggcttctggataccgaaggtaagatgggtcctaattgggcgatttgccactgatacaggaggctactcatgaacgaaagacaattaacaggtgaGATTGTCCCAAATGGTAAACAGAAGGAAATGATTTTCCAGTTCCTg                                                                                                                                                                                                                                                                 >  1:307327‑M2/256‑301 (MQ=255)`  
                                               `gATGGAGACTTTGACATTCTTGCGGTTGTGGTTGACGAAGATGTGCTTATTTTCACATGGGACGAAGTAAAAGGTAAGACATCCATGTGTGTCGGCAAGAGAAACAAAGGCATAAAACTATAGGAGAAATTATTATGGCTATGACAAAGAAATTTAAAGTGTCCTTCGACGTTACCGCAAAGATGTCGTCTGACGTTCAGGCAATCTTAGAGAAAGATATGCTGCATCTATGTAAGCAGGTCGGCTCAGGTGAGATTGTCCCCAATGGTAAACAGAAGGAAATGATTGTCAAGtctgtctt                                                                                                                                                                                                                                                               >  1:576370/1‑294 (MQ=255)`  
                                                    `ggcttgaagcatggctgggatggggactttgcgtacatggcatgggtacatgatgaaatccaagtaggctgccgtaccgaatagattgctcaggtggtcattgagaccgcacaagaagcgatgcgctgggttggagaccactggaacttccggtgtcttctggataccgaaggtaagatgggtcctaattggtcgatttgccactgatacaggaggctactcatgaacgaaagacacttaacaggtgcGATTGTCCCCAATGGTAAACAGAAGGAAATGATTGTCCAGTTCCTGACACACg                                                                                                                                                                                                                                                          >  1:553359‑M2/249‑301 (MQ=255)`  
                                                    `ggcttgaagcatggctgggatggggactttgcgtacatggcatgggtacatgatgaaatccaagtaggctgccgtaccgaagagagtgctcaggtggtcattgagatcgcacaagaagcgatgcgctgggttggagaccattggaacttccggtgtcttctggataccgacggtaagatgggtcataattgggcgatttgccactgatacaggaggctactcatgaaaaaaagacacttaacaggtgcGATTGTCACCAATGGTACACAGAAGGAAAGGATTGTACagttcctgtctctaa                                                                                                                                                                                                                                                          >  1:70344‑M2/249‑294 (MQ=255)`  
                                                     `gaCTTTGACATTCTTGCGGTTGTGGTTGACGAAGATGTGCTTATTTTCACATGGGACGAAGTAAAAGGTAAGACATCCATGTGTGTCGGCAAGAGAAACAAAGGCATAAAACTATAGGAGAAATTATTATGGCTATGACAAAGAAATTTAAAGTGTCCTTCGACGTTACCGCAAAGATGTCGTCTGACGTTCAGGCAATCTTAGAGAAAGATATGCTGCATCTATGTAAGCAGGTCGGCTCAGGTGCGATTGTCCCCAATGGTAAACAGAAGGAAATGATTGTCCAGTTCCTGACACACgg                                                                                                                                                                                                                                                         >  1:189620/1‑301 (MQ=255)`  
                                                         `gaagcatggctgggatggggactttgcgtacatggcatgggtacatgatgaaatccaagtaggctgccgtaccgaagagattgctcaggtggtcattgagaccgcacaagaagcgatgcgctgggttggagaccactggaacttccggtatcttctggataccgaaggtaagatgggtcctaattgggcgatttgccactgatacaggaggctactcatgaacgaaagacacttaacaggtgcGATTGTCCCCAATGGTAAACAGAAGGAAATGATTGTCCAGTTCCTGACACACGGTATg                                                                                                                                                                                                                                                     >  1:495872‑M2/244‑301 (MQ=255)`  
                                                             `gctggctgagaaggggactttgcgtacatagcatggggaaattatgaagtgcaagaaggctgccgtactgaagagattggtcaggtgggcattgagaccgcacaagaagcgatgcgctgggttggagaacactggaacttccggtttcttctgggtaccgaaggtaagatgggtgctaattgggcgatttgccactgatacagaaggctactaatgaacgaaagacacttaacaggtgcGATTGTCCCCAATGGTAAACAGAAGGAAATGATTGTCCAGTTCCTGACACACGGTATGGAAg                                                                                                                                                                                                                                                 <  1:325933‑M2/62‑1 (MQ=255)`  
                                                             `catggctgggatggggactttgcgtacatggcatgggtacatgatgaaatccaagtaggctgccgtaccgaagagattgctcaggtggtcattgagaccgcacaagaagcgatgcgctgggttggagaccactggaacttccggtgtcttctggataccgaaggtaagatgggtcctaatttggcgatttgccactgatacaggaggctactcatgaacgaaagacacttaacaggtgcGATTGTCCCCAATGGTAAACAGAAGGAAATGATTGTCCAGTTCCTGACACACGGTATGGAAg                                                                                                                                                                                                                                                 <  1:48424‑M2/62‑1 (MQ=255)`  
                                                             `aatggatgggatggggactttgcgtacatggcatgggtacatgatgaaatccaagtaggctgccgtaccgaagagattgctcaggtggtcattgagaccgcacaagaagcgatgcgctgtggtggagaccactggaacttccggtgtcttctggataccgaaggtaagatgggtcctaattgggcgatttgccactgatacaggaggctactcatgaacgaaagacacttaacaggtgcGATTGTCCCCAATGGTAAACAGAAGGAAATGATTGTCCAGTTCCTGACACACGGTATGGAAg                                                                                                                                                                                                                                                 <  1:548941‑M2/62‑1 (MQ=255)`  
                                                              `atggctggtatggtgactttgcgtacatggcatgggtacatgatgaaagccaagtaggctgccggaccgaagagattgctcaggtggtcattgagcccgcacaagaagcgatgcgctgggttggagaccactggaacttccggtgtcttctggataccgaaggtaagatgggtcctaattgggcgatttgccactgatacaggaggctactcatgaacgaaagacacttaacaggtgcGATTGTCCCCAATGGTAAACAGGAGGAAATGATTGTCCAGTTCCTGACACACGGTATGGAAgg                                                                                                                                                                                                                                                <  1:118020‑M2/63‑1 (MQ=255)`  
                                                              `atggctgggatggggactttgcgtacatggcatgggtacatgatgaaatccgagtaggctgccgtaccgaagagattgctcaggtggtcattgagaccgcacaagaagcgatgcgctgggttggagaccactggaacttccggtgtcttctgtataccgaaggtaagatgggtcctaattgggcgatttgccactgatacaggaggctactcatgaacgaaagacacttaacaggtgcGATTGTCCCCAATGGTAAACAGAAGGAAATGATTGTCCAGTTCCTGACACACGGTATGGAAgg                                                                                                                                                                                                                                                <  1:380311‑M2/63‑1 (MQ=255)`  
                                                              `atggctgggatggggactttgcgtacagggcatgggtacatgatgaaagccaagtaggctgccgtaccgaagagattgctcaggtggtaattgagaccgcacaagaagcgatgcgctgggttggagaccactggaacttccggtgtcttctggataccgaaggtaagatgggtcctaattgggcgatttgccactgatacaggaggctactcatgaacgaaagacacttaacaggtgcGATTGTCCCCAATGGTAAACAGAAGGAAATGATTGTCCAGTTCCTGACACACGGTATGGAAgg                                                                                                                                                                                                                                                <  1:594643‑M2/63‑1 (MQ=255)`  
                                                               `tggctgggatggggactttgcgtacatggcatgggtacatgatgaaatccaagtaggctgccgtaccgaagagattgctcaggtggtcattgagaccgcacaagaagcgatgtgcttggttggagaccactggaacttccggtgtcttctggataccgaaggtaagatgggtcctaattgggcgatttgccactgatacaggaggctactcatgaacgaaagacacttaacaggtgcGATTGTCCCCAATGGTAAACAGAAGGAAATGATTGTCCAGTTCCTGACACACGGTATGGAAGGa                                                                                                                                                                                                                                               >  1:338427‑M2/238‑301 (MQ=255)`  
                                                                          `gggactttgcgtacatggcatgggtacatgatgaaatccaagtaggctgccgtaccgaagagattgctcaggtggtcattgagaccgcacaagaagcgatgcgctgggttggagaccactggaacttccggtgtcttctggataccgaaggtaagatgggtcctaattgggcgatttgccactgatacaggaggctactcatgaacgaaagacacttaacaggtgcGATTGTCCCCAATGGTAAACAGAAGGAAATGATTGTCCAGTTCCTGACACACGGTATGGAAGGATTGATGACAtt                                                                                                                                                                                                                                    >  1:274003‑M2/227‑301 (MQ=255)`  
                                                                               `tGACGAAGATGGTCTTATTTTCACATGGGACGAAGTAAAAGGTAAGACATCCATGTGTGTCGGCAAGAGAAACAAAGGCATAAAACTATAGGAGAAATTATTATGGCTATGACAAAGAAATTTAAAGTGTCCTTCTACGTTACCGCAAAGAAGTCGTCTGACGTTCAGGCAATCTTAGAGAAAGATATGCTGCATCTATGTAAGCAGGTCGGCTCAGGTGCGATTGTCCCCAATGGTAAACAGAAGGAAATGATTGTCCAGTTCCTGACACACGGTATGGAAGGATTGATGACATTCgtag                                                                                                                                                                                                                               <  1:42806/301‑1 (MQ=255)`  
                                                                                `ttgcgtacatggcatgggtacatgatgaaatccaagtaggctgccgtaccgaagagattgctcaggtggtcattgagaccgcacaagaagcgatgcgctgggttggagaccactggaacttccggtgtcttctggataccgaaggtaagatgggtcctaattgggcgatttgccactgatacaggaggctactcatgaacgaaagacacttaacaggtgcGATTGTCCCCAATGGTAAACAGAAGGAAATGATTGTCCAGTTCCTGACACACGGTATGGAAGgattgatgacctgtctctt                                                                                                                                                                                                                              >  1:141528‑M2/221‑292 (MQ=255)`  
                                                                                `gACGAAGATGTGCTTATTTTCACATGGGACGAAGTAAAAGGTAAGACATCCATGTGTGTCGGCAAGAGAAACAAAGGCATAAAACTATAGGAGAAATTATTATGGCTATGACAAAGAAATTTAAAGTGTCCTTCGACGTTACCGCAAAGATGTCGTCTGACGTCCAGGCAATCTTAGAGAAAGATATGCTGCATCTATGTAAGCAGGTCGGCTCAGGTGCGATTGTCCCCAATGGTAAACAGAAGGAAATGATTGTCCAGTTCCCGACACACGGTATGGAAGGATTGATGACATTCgtagt                                                                                                                                                                                                                              <  1:281358/301‑1 (MQ=255)`  
                                                                                 `aCGAAGATGTGCTTATTTTCACATGGTACGAAGTAAAAGGTAAGACATCCATGTGTGTCGGCAAGAGAAACAAAGGCATAAAACTATAGGAGAAATTATTATGGCTATGACAAAGTAATTTAAAGTGTTCTTCGACGTTACCGCAAAGATGTCGTCTGACGTTCAGGCAATCTTAGAGAAAGATATGCTGCATCTATGTAAGCAGGTCGGCTCAGGTTCGATTGTCCCACATGGTAAACAGAAGGAAATGATTGTCCAGTTCCTGACCCCCGGTATGGCAGGTTtgatgctgtcctttata                                                                                                                                                                                                                             >  1:332814/1‑289 (MQ=255)`  
                                                                                 `aCGAAGATGTGCTTATTTTCACATGGGACGAAGTAAAAGGTAAGACATCCATGTGTGTCGGCAAGAGAAACAAAGGCATAAAACTATAGGAGAAATTATTATGGCTATGACAAAGAAATTTAAAGTGTCCTTCGACGTTACCGCAAAGATGTCGTCTGACGTTCAGGCAATCTTAGAGAAAGATATGCTGCATCTATGTAAGCAGGTCGGCTCAGGTGCGATTGTCCCCAATGGTAAACAGAAGGAAATGATTGTCCAGTTCCTGACACACGGTATGGAAGGATTGATGACATTCgtagta                                                                                                                                                                                                                             <  1:219024/301‑1 (MQ=255)`  
                                                                                        `catggcatgggtacatgatgaaatccaagtaggctgccgtaccgaagagattgctcaggtggtcattgagaccgcacaagaagcgatgcgctgggttggagaccactggaacttccggtgtcttctggataccgaaggtaagatgggtcctaattggcgatttgccactgatacaggaggctactcatgaacgaaagacacttaacaggtgcGATTGTCCCCAATGGTAAACAGAAGGAAATGATTGTCCAGTTCCTGACACACGGTATGGAAGGATTGATGAAATTCGTAGTACGTAcat                                                                                                                                                                                                                      >  1:367962‑M2/213‑301 (MQ=255)`  
                                                                                                 `gtacatgatgaaatccaagtaggctgccgtaccgaagagattgctcaggtggtcattgagaccgcacaagaagcgatgcgctgggttggagaccactggaacttccggtgtcttctggataccgaaggtaagatgggtcctaattgggcgatttgccactgatacaggaggctactcatgaacgaaagacacttaacaggtgcGATTGTCCCCAATGGTAAACAGAAGGAAATGATTGTCCAGTTCCTGACACACGGTATGGAAGGATTGATGACATTCGTAGTACGTACATCATTTCGTg                                                                                                                                                                                                             >  1:269281‑M2/204‑301 (MQ=255)`  
                                                                                                      `tgatgaaatccaagtaggctgccgtaccgaagagattgctcaggtggtcattgagaccgcacaagaagcgatgcgctgggttggagaccactggaacttccggtgtcttctggataccgaaggtaagatgggtcctaattgggcgatttgccactgatacaggaggctactcatgaacgaaagacacttaacaggtgcGATTGTCCCCAATGGTAAACAGAAGGAAATGATTGTCCAGTTCCTGACACACGGTATGGAAGGATTGCTGACATTCGTAGTACGTACATCATTTCGTGAGGcc                                                                                                                                                                                                        >  1:479182‑M2/199‑301 (MQ=255)`  
                                                                                                            `aatccaagtaggctgccgtaccgaagagattgctcaggtggtcattgagaccgcacaagaagcgatgcgctgggtttgataccactggaacttccggtgtcttctggataccgaaggtaagatgggtcctaagtgggcgatttgccactgatacaggaggctactcatgaacgaaagtcacttaacaggtgcGATTGTCCCCAATGGTAAACAGAAGGAAATGATTGTCCAGTTCCTGACACACGGTATGGAAGGATTGATGACATTCGTAGTACGTACATCATTTCGTGAGGCAATTAAg                                                                                                                                                                                                  <  1:569876‑M2/109‑1 (MQ=255)`  
                                                                                                              `tccaagtaggctgccgtaccgaagagattgctcaggtggtcattgagaccgaacaagaagcgatgcgctgggttggagaccactggaacttccggtgtcttctggataccgaaggtaagatgggtcctaattgggcgatttgccactgatacaggaggctactcatgaacgaaagacacttaacaggtgcGATTGTCCCCAATGGTAAACAGAAGGAAATGATTGTCCAGTTCCTGACACACGGTATGGAAGGATTGATGACATTCGTAGTACGTACATCATTTCGTGAGGCCATTAAGGa                                                                                                                                                                                                <  1:400391‑M2/111‑1 (MQ=255)`  
                                                                                                               `ccaagtaggctgccgtaccgaagagattgctcaggtggtcattgagaccgcacaagaagcgatgcgctgggttggagaccactggaacttccggtgtcttctggataccgaaggtaagatgggtcctaattgggcgatttgccactgatacaggaggctactcatgaacgaaagacacttaacaggtgcGATTGTCCCCAATGGTAAACAGAAGGAAATGATTGTCCAGTTCCTGACACACGGTATGGAAGGATTGATGACATTCGTAGTACGTACATCATTTCGTGAGGCCATTAAGGAc                                                                                                                                                                                               <  1:66778‑M2/112‑1 (MQ=255)`  
                                                                                                               `aaGTAAAAGGTAAGACATCCATGTGTGTCGGCAAGAGAAACAAAGGCATAAAACTATAGGAGTAATTATTATGGCTATGACAAAGAAATTTAAAGTGTCCTTCGACGTTACCGCAAAGATGTCGTCTGACGTTCAGGCAATCTTAGAGAAAGATATGCTGCATCTATGTAAGCAGGTCGGCTCAGGTGCGATTGTCCCCAATGGTAAACAGAAGGAAATGATTGTCCAGTTCCTGACACACGTTATGGAAGGATTGATGACATTCGTAGTACGTACATCATTTCGTGAGGCCATTAAGGAc                                                                                                                                                                                               <  1:9291/301‑1 (MQ=255)`  
                                                                                                               `aaGTAAAAGGTAAGACATCCATGTGTGTCGGCAAGAGAAACAAAGGCATAAAACTATAGGAGAAATTATTATGGCTATGACAAAGAAATTTAAAGTGTCCTTCGACGTTACCGAAAAGATGTCGTCTGACGTTCAGGCAATCTTAGAGAAAGATATGCTGCATCTATGTAAGCAGGTCGGCTCAGGTGCGATTGTCCCCAATGGTAAACAGAAGGAAATGATTGTCCAGTTCCTGACACACGGTATGGAAGGATTAATGACATTCGTAGTACGTACATCCTTTCGTgctgtctcttataca                                                                                                                                                                                               >  1:655577/1‑287 (MQ=255)`  
                                                                                                                `caagtaggctgccgtaccgaagagattgctcaggtggtcattgagaccgcacaagaagcgatgcgctgggttggagaccactggaacttccggtgtcttctggataccgaaggtaagatgggtcctaattgggcgatttgccactgatacaggaggctactcatgaacgaaagacacttaacaggtgcGATTGTCCCCAATGGTAAACAGAAGGAAATGATTGTCTAGTTCCTGACACACGGTATGGAAGGATTGATGACATTCGTAGTACGTACATCATTTCGTGAGGCCATTACGGACa                                                                                                                                                                                              >  1:442150‑M2/189‑301 (MQ=255)`  
                                                                                                                 `gTAAAAGGTAAGACATCCATGTGTGTCGGCAAGAGAAACAAAGGCATAAAACTATAGGAGAAATTATTATGGCTATGACAAAGAAATTTAAAGTGTCCTTCGACGTTACCGCAATGATGTCGTCTGACGTTCAGGCAATCTTAGAGAAAGATATGCTGCATCTATGTAAGCAGGTCGGCTCAGGTGCGATTGTCCCCAATGGTAAACAGCAGGAAATGATTGTCCAGTTCCTGACACACGGTATGTAAGGATTGATGACATTCGTAGTACGTACATCATTTCGTgctgtctcttataccca                                                                                                                                                                                             >  1:128578/1‑285 (MQ=255)`  
                                                                                                                    `ataagagccagaccgaagagattgctcaggtggtcattgagaccgcacaagaagcgatgcgctgggttggagaccactggaacctccggtgtcttctggataccgaaggtaagatgggtcctaattgggcgatttgccactgatacaggaggctactcatgaacgaaagacacttaacaggtgtGATTGTCCCCAATGGTAAACAGAAGGAAATGATTGTCCAGTTCCTGACACACGGTATGGAAGGATTGATGACATTCGTAGTACGTACATCATTTCGTGAGGACATTAAGGACATGCa                                                                                                                                                                                          <  1:257757‑M2/117‑1 (MQ=255)`  
                                                                                                                       `ggTAAGACATCCATGTGTGTCGGCAAGAGAAACAAAGGCATAAAACTATAGGAGAAATTATTATGGCTATGACAAAGAAATTTAAAGTGTCCTTCGACGTTACCGCAAAGATGTCGTCTGACGTTCAGGCAATCTTAGAGAAAGATAGGCTGCATCTATGTAAGCAGGTCGGCTCAGGTGCGATTGTCCCCAATGGTAAACAGAAGGAAATGATTGTCCAGTTCCTGACACACGGTATGGAAGGATTGATGACATTCGTAGTACGTACATCATTTCGTGAGGCCATTAAGGACATGCActg                                                                                                                                                                                       >  1:213104/1‑299 (MQ=255)`  
                                                                                                                                `accATTTGTGTCGGCAAGAGAAACAAAGGGATAAAAATATAGGAGAAAGTATTATGGCTATGAGAAAGAAATTTAAAGTGTCCTTCGACGTTACCGCAAAGATGTCGTCTGACGTTCAGGCAATCTTAGAGAAAGATATGCTGCATCTATGTAAGCAGGTCGGCTCAGGTGCGATTGTCCCCAATGGTAAACAGAAGGAAATGATGGTCCAGTTCCTGACACACGGTATGGAAGGATTGATGACATTCGTAGTACGTACATCATATCGTGAGGCCATTAAGGACATGCACGAAGAGTATGc                                                                                                                                                                              <  1:601494/300‑1 (MQ=255)`  
                                                                                                                                  `cATTTGTTTCGGCAAGAGAAACAAAGGCATAAAACTTTAGTAGAAATTATTATAGCAATGACAAGAAAATTTAAAGTGTCCTTCGACGTTACCGCAAAGATGTCGTCTGACGTTCAGGCAATCTTAGCGAAAGATATGCTGCAGCTATGTAAGCAGGTCGGCTCAGGTTCGATTGTCCCCAATGTTAAACAGAAGGAAATGATTGTCCAGTTCCTGACACACGGTATGGAAGGATTGATGACATTCGTAGTACGTACATCATTTCGTGAGGCCATTAAGGACATGCACGAAGAGTATGCAg                                                                                                                                                                            <  1:362953/301‑1 (MQ=255)`  
                                                                                                                                      `agatagctcaggtggtcattgagaccgcacaagaagcgatgcgctgggttggagaccactggaacttccggtgtcttctggataccgaaggtaagatgggtcctaattgggcgatttgccactgatacaggaggctactcatgaacgaaagacactgaacaggtgcGATTGTCCCCAATGGTAAACAGAAGGAAATGATTGTCCAGTTCTTGACACACGGTATGGAAGGATTGATGACATTCGTAGTACGTACATCATTTCGTGAGGCCATTAAGGACATGCACGAAGAGTATGCAGATaa                                                                                                                                                                        >  1:616993‑M2/167‑301 (MQ=255)`  
                                                                                                                                       `gtgtCGGTGAGAGAAACAAAGGCATAAAACTATAGGAGAAATTCTTATGGCTATGACAAAGAAATTTAAAGTGTCCTTCGACGTTACCGCAAAGATGTCGTCTGACGTTCAGGAAATCTTAGAGAAAGATATGCTGCATCTATGTAAGCAGGTCGGCTCAGGTGCGATTGTCCCCAAGGGTAAACAGAAGGAAATGATTGTCCAGTTCCTGACACACGGTATGTCAGGATTGACGACATTAGTAGTACGTACATCATTTCTTGAGGCCATTAAGGACATGAACGAGGAGTATGCAGATAAg                                                                                                                                                                       >  1:520241/1‑301 (MQ=255)`  
                                                                                                                                          `tgctcagttggtcattgggaccgcacaagaagcgatgcgctaggttggagaccacttgaacttccggtgtcttcaggataccgaaggtaagatgggtcctaattgggcgatttgccactgatacaggaggctactcatgaacgaaagacacttaacaggtgcGATTGTCCCCAATGGTAAACAGAAGGAAATGATTGTCCAGTTCCTGACACACGGTATGGAAGGATTGATGACATTCGTAGTACGTACATCATTTCGTGAGGCCATTAAGGACATGCACGAAGAGTATGCAGATAAGGAc                                                                                                                                                                    <  1:636941‑M2/139‑1 (MQ=255)`  
                                                                                                                                           `gctcaggtggtcattgagaccgcacaagaagcgatgcgctgggttggagaccactggaacttccggtgtcttctggataccgaaggtaagatgggtcctaattgggcgatttgccactgatacaggaggctactcatgaacgaaagacacttaacaggtgcGATTGTCCCCAATGGTAAACAGAAGGAAATGATTGTCCAGTTCCTGACACACGGTATGGAAGGATTGATGACATTCGTAGTACGTACATCATTTCGTGAGGCCATTAAGGACATGCACGAAGAGTATGCAGAtaaggctg                                                                                                                                                                   >  1:386655‑M2/162‑298 (MQ=255)`  
                                                                                                                                           `gcgaaggtggtcattgagaccgaacaagaagcgatgcgatgggttgtagacaactggaacttcctgtgtattctggataccgaaggtaagatgggtcctaattgggcgatttgccactgatacaggaggctactcatgaacgaaagacacttaacaggtgcGATTGTCCCCAATGGTAAACAGAAGGAAATGATTGTCCAGTTCCTGACACACGGTATGGAAGGATTGATGACATTCGTAGTACGTACATCATTTCGTGAGGCCATTAAGGACATGCACGAAGAGTATGCAGATAAGGAct                                                                                                                                                                   <  1:350724‑M2/140‑1 (MQ=255)`  
                                                                                                                                                `ggtggtcattgagaccgcacaagaagcgatgcgctgggttggagaccactggaacttccggtgtcttctggataccgaaggtaagatgggtcctaattgggcgatttgccactgatacaggaggctactcatgaacgaaagacacttaacaggtgcGATTGTCCCCAATGGTAAACAGAAGGAAATGATTGTCCAGTTCCTGACACACGGTATGGAAGGATTGATGACATTCGTAGTACGTACATCATTTCGTGAGGCCATTAAGGACatgcacgaagagtatgcctgtctcttatacaca                                                                                                                                                              >  1:501999‑M2/157‑285 (MQ=255)`  
                                                                                                                                                    `gtcattgagaccgcacaagaagcgatgcgctgggttggagaccactggaacttccggtgtcttctggataccgaaggtaagatgggtcctaattgggcgatttgccactgatacaggaggctactcatgaacgaaagacacttaacaggtgcGATTGTCCCCAATGGTAAACAGAAGGAAATGATTGTCCAGTTCCTGACACACGGTATGGAAGGATTGATGACATTCGTCGTACGTACATCATTTCGTGAGGCCATTAAGGACATGCACGAAGAGTATGCAGCTACGGACTCTTTcaaaa                                                                                                                                                          >  1:423090‑M2/153‑300 (MQ=255)`  
                                                                                                                                                           `ggCATAAAACTATAGGAGAAATTATTATGGCTATGACAAAGAAATTTAAAGTGTCCTTCGACGTTACCGCAAAGATGTCGTCTGACGTTCAGGCAATCTTAGAGACAGATATGCTGCATCTATGTAAGCAGGTCGGCTCAGGTGCGATTGTCCCCAATGGTAAACAGAAGGAAATGATTGTCCAGTTCCTGACACACGGTATGGAAGGATTGATGACATTCGTAGTACGTACATCATTTCGTGAGGCCATTAAGGACATGCACGAAGAGTATGCAGATAAGGACTCTTTCAAACAATCTcc                                                                                                                                                   >  1:46740/1‑301 (MQ=255)`  
                                                                                                                                                                   `aCTATAGGAGAAATTATTATGGCTATGACAAAGAAATTTAAAGTGTCCTTCGACGTTACCGCAAAGATGTCGTCTGACGTTCAGGCAATCTTAGAGAAAGATATGCTGCATCTATGTAAGCAGGTCGGCTCAGGTGCGATTGTCCCCAATGGTAAACAGAAGGAAATGAATGTCCAGTTCCTGACACACGGTATGGAAGGATTGTTGACATTCGTAGTACGTACATCATTTCGTGAGGCCATTAAGGACATGCATGAATAGTATGCAGATATGGACTCTTCCAAACAATTTCCTGCAACAg                                                                                                                                           >  1:649101/1‑301 (MQ=255)`  
                                                                                                                                                                    `cTATAGGAGAAATTATTATGGCTATGACAAAGAAATTTAAAGTGTCCTTCGACGTTACCGCAAAGATGTCGTCTGACGTTCAGGCAATCTTAGAGAAAGATATGCTGCATCTATGTAAGCAGGTCGGCTCAGGTGCGATTGTCCCCAATGGTAAACCGAAGGAAATGATTGTCCAGTTCCTGACACACGGTATGGATGGATTGATGACATTCGTAGTACGTACATCATTTCGTGAGGCCATTAAGGACATGCACGAAGAGTATGCAGATAAGGACTCTTTCACACACTATCCTGCAACAGt                                                                                                                                          >  1:453515/1‑301 (MQ=255)`  
                                                                                                                                                                         `gcgatgcgctgggttggagaccactggaacttccggtgtcttctggataccgaaggtaagatgggtcctaattgggcgatttgccactgatacaggaggctactcatgaacgaaagacacttaacaggtgcGATTGTCCCCAATGGTAAACAGAAGGAAATGATTGTCCAGTTCCTGACACACGGTATGGAAGGATTGATGACATTCGTAGTACGTACATCATTTCGTGAGGCCATTAAGGACATGCACGAAGAGTATGCAGATAAGGACTCTTTCAAACAATCTCCTGCAACAGTACggg                                                                                                                                     >  1:543484‑M2/132‑301 (MQ=255)`  
                                                                                                                                                                             `tgcgctgggttggagaccactggaacttccggtgtcttctggataccgaaggtaagatgggtcctaattgggcgatttgccactgatacaggaggctactcatgaacgaaagacacttaacaggtgcGATTGTCCCCAATGGTAAACAGAAGGAAATGATTGTCCAGTTCCTGACACACGGTATGGAAGGATTGATGACATTCGTAGTACGTACATCATTTCGTGAGGCCATTAAGGACATGCACGAAGAGTATGCCGATAAGGACTCTTTCAAACAATATCCTGCAACAGTACGGGAGgt                                                                                                                                 >  1:100060‑M2/128‑301 (MQ=255)`  
                                                                                                                                                                              `aaTTATTATGGCTATGACAAAGAAATTTAAAGTGTCCTTCGACGTTACCGCAAAGATGTCGTCTGACGTTCAGGCAAGCTTAGAGAAAGATATGCTGCATCTATGTAAGCAGGTCGGCTCAGGTGCGATTGTCCCCAATGGTAAACAGAAGGAAATGATTGTCCAGTTCCTGACACACGGTATGGAAGGATTGATGACATTCTTAGTACGTACATCATTTCGTGAGGCCATTAAGGACATGCACGAAGAGTATGCAGATAAGGACTCTTTCAAACAATCTCCTGCAACAGTACGGGAGgtg                                                                                                                                <  1:252057/301‑1 (MQ=255)`  
                                                                                                                                                                                   `gggttggagaccactggaacttccggtgtcttctggataccgaaggtaagatgggtcctaattgggcgatttgccactgatacaggaggctactcatgcacgaaagacacttaacaggtgcGATTGTCCCCAATGGTAAACAGAAGGAAATGATTGTCCAGTTCCTGACACACGGTATGGAAGGATTGATGACATTCGTAGTACGTACATCATTTCGTGAGGCCATTAAGGACATGCACGAAGAGTATGCAGATAAGGACTCTTTCAAACAATCTCCTGCAACAGTACGGGAGGTGTTCTg                                                                                                                           >  1:245348‑M2/122‑301 (MQ=255)`  
                                                                                                                                                                                   `gggttggagaccactggaacttccggtgtcttctggataccgaaggtaagatgggtcctaattgggcgatttgccactgatacaggaggctactcatgaacgaaagacacttaacaggtgcGATTGTCCCCAATGGTAAACAGAAGGAAATGATTGTCCAGTTCCTGACACACGGTATGGAAGGA‑TGATGACATTCGTAGTACGTACATCATTTCGTGAGGCCATTAAGGACATGCACGAAGAGTATGCAGATAAGGACTCTTTCAAACAATCTCCTGCAAAAGTACGGGAGGTGTTCTGa                                                                                                                          >  1:410441‑M2/122‑301 (MQ=255)`  
                                                                                                                                                                                   `gggttggagaccactggaacttccggtgtcttctggataccgaaggtaagatgggtcctaattgggcgatttgccactgatacaggaggctactcatgaacgaaagacacttaacaggtgcGATTGTCCACAATGGTAAACAGAAGGAAATGATTGTCCAGTTCCTGACACACGGTATGGAAGGATTGATGACATTCGTAGTACGTACATCATTTCGTGAGGCCATTAAGTACATGCACGAAGAGTATGCAGATAAGGACTCTTTCAAACAATCTCCTGCAACAGTACGGGAGGTGTTCTg                                                                                                                           >  1:165518‑M2/122‑301 (MQ=255)`  
                                                                                                                                                                                      `ttggagaccactggaacttccggtgtcttctggataccgaaggtaagatgggtcctaattgggcgatttgccactgatacaggaggctactcatgaacgaaagacacttaacaggtgcGATTGTCCCCAATGGTAAACAGAAGGAAATGATTGTCCAGTTCCTGACACACGGTATGGAAGGATTGATGACATTCGTAGTACGTACATCATTTCGTGAGGCCATTAAGGACATGCACGAAGAGTATGCAGATAAGGACTCTTTCAAACAATCTCCTGCAACAGTACGGGAGGTGTTCTGA‑Tg                                                                                                                       <  1:233573‑M2/183‑1 (MQ=255)`  
                                                                                                                                                                                      `tGGCTATGACAAAGAAATTTAAAGTGTCCCTCGACGTTACCGCAAAGATGTCGTCTGACGTTCAGGCAATCTTAGAGAAAGTTATGCTGCATCTATGTAAGCAGGTCGGCTCAGGTGCGATTGTCCCCAATGGTAAACAGAAGGAAATGATTGTCCAGTTCCTGACACACGGTATGGAAGGATTGATGACATTCGTAGTACGTACATCATTTCGTGAGGCCATTAAGGACATGCACGAAGAGTATGCAGATAAGGACTCTTTCAAACAATCTCCTGCAACAGTACGGGAGGTGTTCTGA‑Tg                                                                                                                       <  1:44757/301‑1 (MQ=255)`  
                                                                                                                                                                                                     `aaTTTAAAGTGTCCTTCGACGTTACCGCAAAGATGTCGTCTGACGTTCAGGCAATCTTAGAGAAAGATATGCTGCATCTATGTAAGCAGGTCGGCTCAGGTGCGATTGTCCCCAATGGTAAACAGAAGGAAATGATTGTCCAGTTCCTGACACACGGTATGGAAGGATTGATGACATTCGTAGTACGTACATAATTTCGTGAGGCCATTAAGGACATGCACGAAGAGTATGCAGATAAGGACTCTTTCAAACAATCTCCTGCAACAGTACGGGAGGTGTTCTGACTGTCtcttatacacat                                                                                                         >  1:75658/1‑290 (MQ=255)`  
                                                                                                                                                                                                      `agttAAAGTGTCCTTCGACGTTACCGCAAAGATGTCGTCTGACGTTCAGGCAATCTTAGAGAAAGATATGCTGCATCTATGTAAGCAGGTCGGCTCAGGTGCGATTGTCCCCAATGGTAAACAGAAGGAAATGATTGTCCAGTTCCTGACACACGGTATGGAAGGATTGATGACATTCGTAGTACGTACATCATTTCGTGAGGCCATTAAGGACATGCACGAAGAGTATGCAGATAAGGACTCTTTCAAACAATCTCCTGCAACAGTACGGGAGGTGTTCTGA‑TGTCTGACTACCTGAAAg                                                                                                       <  1:201037/299‑1 (MQ=255)`  
                                                                                                                                                                                                       `ttccggtgtcttatggataccgaaggtaagatgggtcctaattgggcgatttgccactgatacaggaggctactcatgaacggaagacacttaacaggtgcGATTGTCCCCAATGGTAAACAGAAGGAAATGATTGTCCAGTTCATGACACACGGTATGGAAGGATTGATGACATTCGTAGTACGTACATCATTTCGTGAGGCCATTAAGGACATGCACGAAGAGTATGCAGATAAGGACTCTTTCAAACAATCTCCTGCAACAGTACGGGAGGTGTTCTGA‑TGTCTGACTACCTGAAAGt                                                                                                      <  1:22644‑M2/200‑1 (MQ=255)`  
                                                                                                                                                                                                       `tgtAAAGTGTCCTTCGACGTTACCGCAAAGATGTCGTCTGACGTTCAGGCAATCTTAGAGAAAGATATGCTGCATCTATGTAAGCAGGTCGGCTCAGGTGCGATTGTCCCCAATGGTAAACAGAAGGAAATGATTGTCCAGTTCCTGACACACGGTATGGAAGGATTGATGACATTCGTAGTACGTACATCATTTCGTGAGGCCATTAAGGACATGCACGAAGAGTATGCAGATAAGGACTCTTTCAAACAATCTCCTGCAACAGTACGGGAGGTGTTCTGA‑TGTCTGACTACCTGAAAGt                                                                                                      <  1:302294/299‑1 (MQ=255)`  
                                                                                                                                                                                                             `gtgtCCTTCGACGTTACCGCAAAGATGTCGTCTGACGTTCAGGCAATCTTAGAGAAAGATATGCTGCATCTATGTAAGCAGGTCGGCTCAGGTGCGATTGTCCCAAATGGTAAACAGAAGGAACTGAATGTCCAGTTCCTGACACCCGGTATGGAAGGATTGATGACATTCGTAGTACGTACATCATTTCGTTAGGCCATTAAGTACATGCACGAAGAGTATGCAGATAAGGTCTCTTTCAAACAATCTCCTGCATCAGTACGGGAGGTGTTCTGA‑TGTCTGACTCCCTGACAGtcctgcc                                                                                                >  1:81593/1‑300 (MQ=255)`  
                                                                                                                                                                                                               `tcgtattgataccgaagtaaagctgtatcctaattgggcgatttgccactgatacaggaggctactcatgaacgacagacacttaacaggtgcGATTGTCCCCAATGGTAACCAGAATGAAATGATTGTCCAGTTCCTGACACACGGTATGGAAGGATTGATGACATTCGTAGTACGTACATCATTTCGTGATGCCATTAAGGACATGCACGAATAGTATGCAGATAAGGACTCTTTCAAACAATCTCCTGCAACAGTACGGGAGGTGTTCTGA‑TGTCTGACTACCTGAAAGTGCTgcaag                                                                                              <  1:239134‑M2/208‑1 (MQ=255)`  
                                                                                                                                                                                                                         `accgaaggtaagatgaggcctgattgggcgatttaacactgatacaggaagctactaatgaacgaaagacacttagcaggtgcGATTGTCCCGAATGGTAAACAGAAGGAAATGATTGACCGGTTCCTGACACACGGTATGGAAGGATTGATGACATTCGTAGTACGTACATCGTTACGTGAGGCCATTAAGGACATGCACGAAGAGTATGCAGATAAGGACTCTTTCAAACAATCTCCTACAACAGTACGGGAAGTGTTCTGA‑TGTCTGACTACCTGAAAGTGCTGCAAGCAATCAAAAg                                                                                    <  1:356845‑M2/218‑1 (MQ=255)`  
                                                                                                                                                                                                                                     `atgggtcctaattgggcgatttgccactgatacaggaggctactcatgaacgaaagacacttaacaggtgcGATTGTCCCCAATGGTAAACAGAAGGAAATGATTGTCCAGTTCCTGACACACGGTATGGAAGGATTGATGACATTCGTAGTACGTACATCATTTCGTGAGGCCATTAAGGACATGCACGAAGAGTATGCAGATAAGGACTCTTTCAAACAATCTCCTGCAACAGTACGGGAGGTGTTCTGA‑TGTCTGACTACCTGAAAGTGCTGCAAGCAATCAAAAGTTGCCCTAAGAc                                                                        <  1:194661‑M2/230‑1 (MQ=255)`  
                                                                                                                                                                                                                                     `aTGTCGTCTGACGTTCAGGCAATCTTAGAGAAAGATATGCTGCATCTATGTAAGCAGGTCGGCTCAGGTGCGATTGTCCCCAATGGTAAACAGAAGGAAATGATTGTCCAGTTCCTGACACACGGTATGGAAGGATTGATGACATTCGTAGTACGTACATCATTTCGTGAGGCCATTAAGGACATGCACGAAGAGTATGCAGATAAGGACTCTTTCAAACAATCTCCTGCAACAGTACGGGAGGTGTTCTGA‑TGTCTGACTACCTGAAAGTGCTGCAAGCAATCAAAAGTTGCCCTAAGAc                                                                        <  1:154976/301‑1 (MQ=255)`  
                                                                                                                                                                                                                                       `gggtcctaattgggcgatttgccactgatacaggaggctactcatgaacgaaagacacttaacaggtgcGATTGTCCCCAATGGTAAACAGAAGGAAATGATTGTCCAGTTCCTGACACACGGTATGGAAGGATTGATGACATTCGTAGTACGTACATCATTTCGTGAGGCCATTAAGGACATGCACGAAGAGTATGCAGATAAGGACTCTTTCAAACAATCTCCTGCAACAGTACGGGAGGTGTTCTGA‑TGTCTGACTACCTGAAAGTGCTGCAAGCAATCAAAAGTTGCCCTAAGACtt                                                                      >  1:404633‑M2/70‑301 (MQ=255)`  
                                                                                                                                                                                                                                          `tcctaattgggcgatttgccactgatacaggaggctactcatgaacgaaagacacttaacaggtgcTCTTGTCCCCAATGGTAAACAGACGGAAATGATTGTCCAGTTCCTGACACACGGTATTGAAGGATTGATGACATTCTTATTACGTACATCATTTCGTGAGGCCATTAAGGACATGCACGAAGCGTATGCAGATAAGGACTCTTTCAAACAATCTCCTGCAACAGTACGGGAGGTGTTCTGA‑TGTCTGACTACCTGAAAGTGCTGCAAGCAATCACAAGTTGCCCTAAGACTTTcc                                                                   >  1:660906‑M2/67‑301 (MQ=255)`  
                                                                                                                                                                                                                                          `ggagatgtgtaaaagagacagctgatacaggaggctactcatgaacgaaagacacttaacaggtgcGATTGTCCCCAATGGTAAACAGAAGGAAATGATTGTCCAGTTCCTGACACACGGTATGGAAGGATTGATGACATTCGTAGTACGTACATCATTTCGTGAGGCCATTAAGGACATGCACGAAGAGTATGCAGATAAGGACTCTTTCAAACAATCTCCTGCAACAGTACGGGAGGTGTTCTGA‑TGTCTGACTACCTGAAAGTGCTGCAAGCAATCAAAAGTTGCCCTAAGACTTTcc                                                                   <  1:490660‑M2/235‑1 (MQ=255)`  
                                                                                                                                                                                                                                                    `gcgatttgccactgatacaggaggctactcatgaacgaaagacacttaacaggtgcGATTGTCCCCAATGGTAAACAGAAGGAAATGATTGTCCAGTTCCTGACACACGGTATGGAAGGATTGATGACATTCGTAGTACGTACATCATTTCGTGAGGCCATTAAGGACATGCACGAAGAGTATGCAGATAAGGACTCTTTCAAACAATCTCCTGCAACAGTACGGGAGGTGTTCTGA‑TGTCTGACTACCTGAAAGTGCTGCAAGCAATCAAAAGTTGCCCTAAGACTTTCCAGTCCAACTa                                                         <  1:515088‑M2/245‑1 (MQ=255)`  
                                                                                                                                                                                                                                                    `aaGGCAATCTTAGAGAAAGATATGCTGCATCTATGTAAGCAGGTCGGCTCAGGTGCGAATGTCCCCAATGGTAAACAGAAGGAAATGATTGTCCAGTTCCTGACACACGGTATGGAAGGATTGATGACATTCGTAGTACGTACATCATTTCGTGAGGCCATTAAGGACATGCACGAAGAGTATGCAGATAAGGACTCTTTCAAACAATCTCCTGCAACAGTACGGGAGGTGTTCTGA‑TGTCTGACTACCTGAAAGTGCTGCAAGCAATCAAAAGTTGCCCTAAGACTTTCCAGTCCAACTa                                                         <  1:149219/300‑1 (MQ=255)`  
                                                                                                                                                                                                                                                              `actgaaagaggtggctaatcatgaactaaagacacttaacaggtgcGATTGTCTCCAATAGTAAACAGAATGAAATGATTGTCCAGTTCCAGACACACGGTATGGATGGATTGATGACATTCGTAGTACGTACATCATTTCGTGAGTCCATTAAGGACATGCACGAAGAGTATGCAGATAAGGACTCTTTCAAACAATCTCCTGCAACAGTACGGGATGTGTTCTGA‑TGTCTGACTACCTGAAAGTGCTGCAAGCAATCAAAAGTTGCCCTAAGACTTTCCAGTCCAACTATGTACGGAAc                                               <  1:631448‑M2/255‑1 (MQ=255)`  
                                                                                                                                                                                                                                                                      `gATATGCTGCATCTATGTAAGCAGGTCGGCTCAGGTGCGATTGTCCCCAATGGTAAACAGAAGGAAATGATTGTCCAGTTCCTGACACACGGTATGGAAGGATTGATGACATTCGTAGTACGTACATCATTTCGTGAGGCCATTAAGGACATGCACGAAGAGTATGCAGATAAGGACTCTTTCAAACAATCTCCTGCAACAGTACGGGAGGTGTTCTGA‑TGTCTGACTACCTGAAAGTGCTGCAAGCAATCAAAAGTTGCCCTAAGACTTTCCAGTCCAACTATGTACGGAAcctgtctct                                       >  1:344015/1‑293 (MQ=255)`  
                                                                                                                                                                                                                                                                       `ggaggctactcatgaacgaaagactcttaacaggtgcGATTGTCCCCAATGGTAAACAGAAGGAAATGATTGTCCAGTTCCTGACACACGGTATGGAAGGATTGATGACATTCGTAGTACGTACATCATTTCGTGAGGCCATTAAGGACATGCACGAAGAGTATGCAGATAAGGACTCTTTCAAACAATCTCCTGCAACAGTACGGGAGGTGTTCTGA‑TGTCTGACTACCTGAAAGTGCTGCAAGCAATCAAAAGTTGCCCTAAGACTTTCCAGTCCAACTATGTACGGAACaatgcctgt                                      >  1:16035‑M2/38‑297 (MQ=255)`  
                                                                                                                                                                                                                                                                         `tcggagatgtgtataagagacagcttaacaggtgcGATTGTCCCCAATGGTAAACAGAAGGAAATGATTGTCCAGTTCCTGACACACGGTATGGAAGGATTGATGACATTCGTAGTACGTACATCATTTCGTGAGGCCATTAAGGACATGCACGAAGAGTATGCAGATAAGGACTCTTTCAAACAATCTCCTGCAACAGTACGGGAGGTGTTCTGA‑TGTCTGACTACCTGAAAGTGCTGCAAGCAATCAAAAGTTGCCCTAAGACTTTCCAGTCCAACTATGTACGAAACAATGCGAGCCt                                    <  1:624906‑M2/266‑1 (MQ=255)`  
                                                                                                                                                                                                                                                                         `aggctactcatgaacgaaagacacttaacaggtgcGATTGTCCCCAATGGTAAACAGAAGGAAATGATTGTCCAGTTCCTGACACACGGTATGGAAGGATTGATGACATTCGTAGTACGTACATCATTTCGTGAGGCCATTAAGGACATGCACGAAGAGTATGCAGATAAGGACTCTTTCAAACAATCTCCTGCAACAGTACGGGAGGTGTTCTGA‑TGTCTGACTACCTGAAAGTGCTGCAAGCAATCAAAAGTTGCCCTAAGACTTTCCAGTCCAACTATGTACGGAACAATGCGAGCCt                                    >  1:642657‑M2/36‑301 (MQ=255)`  
                                                                                                                                                                                                                                                                            `gagatgtgtataagagacagcGGCTCAGGTGCGATTGTCCCCAATGGTAAACAGAAGGAAATGATTGTCCAGTTCCTGACACACGGTATGGAAGGATTGATGACATTCGTAGTACGTACATCATTTCGTGAGGCCATTAAGGACATGCACGAATAGTATGCAGATAAGGACTCTTTCAAACAATCTCCTGCAACAGTACGGGAGGTGTTCTGA‑TGTCTGACTACCTGAAAGTGCTGCAAGCAATCAAAAGTTGCCCTAAGACTTTCCAGTCCAACTATGTACGGAACAATGCGAGCCTCGt                                 <  1:183989/281‑1 (MQ=255)`  
                                                                                                                                                                                                                                                                                   `tATGTAAGCAGGTCGGCTCAGGTGCGATTGTCCCCAATGGTAAACAGAAGGAAATGATTGTCCAGTTCCTGACACACGGTATGGAAGGATTGATGACATTCGTAGTACGTACATCATTTCGTGAGGCCATTAAGGACATGCACGAAGAGTATGCAGATAAGGACTCTTTCAAACAATCTCCTGCAACAGTACGGGAGGTGTTCTGA‑TGTCTGACTACCTGAAAGTGATGCAAGCAATCAAAAGTTGCCCTAAGACTTTCCAGTCCAACTATGTACGGAACAATGCGAGctgtctcttataa                          >  1:558799/1‑289 (MQ=255)`  
                                                                                                                                                                                                                                                                                     `aacgaaagacacttaacaggtgcGATTGTCCCCAATGGTAAACAGAAGGAAATGATTGGCCAGTTCCTGACACACGGTATGGAAGGATTGATGACATTCGTAGTACGTACATCATTTCGTGAGGCCATTAAGGACATGCACGAAGAGTATGCAGATAAGGACTCTTTCAAACAATCTCCTGCAACAGTACGGGAGGTGTTCTGA‑TGTCTGACTACCTGAAAGTGCTGCAAGCAATCAAAAGTTGCCCTAAGACTTTCCAGTCCAACTATGTACGGAACAATGCGAGCCTCGTAGCGGAGGc                        <  1:113136‑M2/278‑1 (MQ=255)`  
                                                                                                                                                                                                                                                                                       `cgaaagacacttaacaggtgcGATTGTCCCCAATGGTAAACAGAAGGAAATGATTGTCCAGTTCCTGACACACGGTATGGAAGGATTGATGACATTCGTAGTACGTACATCATTTCGTGAGGCCATTAAGGACATGCACGAAGAGTATGCAGATAAGGACTCTTTCAAACAATCTCCTGCAACAGTACGGGAGGTGTTCTGA‑TGTCTGACTACCTGAAAGTGCTGCAAGCAATCAAAAGTTGCCCTAAGACTTTCCAGTCCAACTATGTAGGGAACAAGGCGAGCCTAGTAGCGGAGGCCg                      >  1:106587‑M2/22‑301 (MQ=255)`  
                                                                                                                                                                                                                                                                                            `gacacttaacaggtgcGATTGTCCCCAATGGTAAACAGAAGGAAATGATTGTCCAGTTCCTGACACACGGTATGGAAGGATTGATGACATTCGTAGTACGTACATCATTTCGTGAGGCCATTAAGGACATGCACGAAGAGTATGCAGATAAGGACTCTTTCAAACAATCTCCTGCAACAGTACGGGAGGTGTTCTGA‑TGTCTGACTACCTGAAAGTGCTGCAACCAATCAAAAGTTGCCCTAAGACTTTCCAGTCCAACTATGTACGGAACAATGCGAGCCTCGTAGCGGAGGACGCTTcc                 >  1:611981‑M2/17‑301 (MQ=255)`  
                                                                                                                                                                                                                                                                                                  `taacaggtgcGATTGTCCCCAATGGTAAACAGAAGGAAATGATTGTCCAGTTCCTGACACACGGTATGGAAGGATTGATGACATTCGTAGTACGTACATCATTTCGTGAGGACATTAAGGACATGCACGAAGAGTATGCAGATAAGGACTCTTTCAAACAATCTCCTGCAACAGTACGGGAGGTGTTCTGA‑TGTCTGACTACCTGAAAGTGCTGCAAGCAATCAAAAGTTGCCCTAAGACTTTCCAGTCCAACTATGTACGGAACAATGCGAGCCTCGTAGCGGAGGCCGCTTCCCGTGGt           <  1:8808‑M2/291‑1 (MQ=255)`  
                                                                                                                                                                                                                                                                                                   `aacaggtgcGATTGTCCCCAATGGTAATCAGAAGGAAATGATTGTCCAGTTCCTGACACACGGTATGGAAGGATTGATGACATTCGTAGTATGTACATCATTTCGTGAGGCCATTAAGGACATGCACGAAGAGTATGCAGATAAGGACTCTTTCAAACAATCTCCTGCAACAGTACGGGAGGTGTTCTGA‑TGTCTGACTACCTGAAAGTGCTGCAAGCAATCAAAAGTTGCCCTAAGACTTTCCAGTCCAACTATGTACGGAACAATGCGAGCCTCGTAGCGGAGGCCGCttcccgctgtc          >  1:607587‑M2/10‑296 (MQ=255)`  
                                                                                                                                                                                                                                                                                                         `tgcGATTGTCCCCAATGGTAAACAGAAGGAAATGATTGTCCAGTTCCTGACACACGGTATGGAAGGATTGATGACATTCGTAGTACGTACATCATTTCGTGAGGCCATTAAGGACATGCACGAAGAGTATGCAGATAAGGACTCTTTCAAACAATCTCCTGCAACAGTACGGGAGGTGTTCTGA‑TGTCTGACTACCTGAAAGTGCTGCAAGCAATCAAAAGTTGCCCTAAGACTTTCCAGTCCAACTATGTACGGAACAATGCGAGCCTCGTAGCGGAGGCCGCTTCCCGTGGTCACAtct    <  1:564525‑M2/298‑1 (MQ=255)`  
                                                                                                                                                                                                                                                                                                         `ggcGATTGTCCCCAATGGTAAACAGAAGGAAATGATTGTCCAGTTCCTGACACACGGTATGGAAGGATTGATGACATTCGTAGTACGTACATCATTTCGTGAGGCCATTAAGGACATGCACGAAGAGTATGCAGATAAGGACTCTTTCAAACAATCTCCTGCAACAGTACGGGAGGTGTTCTGA‑TGTCTGACTACCTGAAAGTGCTGCAAGCAATCAAAAGTTGCCCTAAGACTTTCCAGTCCAACTATGTACGGAACAATGCGAGCCTCGTAGCGGAGGCCGCTTCCCGTGGTCACAtct    <  1:392250‑M2/298‑1 (MQ=255)`  

                                                                                                                                                                                                                                                                                                            `|`                                                                                                                                                                                                                                                                                                              
`ATGCCAAGCAGGTTAGGCTAGGTGGATGCGGTAGGTCCGAATATAAGGATGGAGACTTTGACATTCTTGCGGTTGTGGTTGACGAAGATGTGCTTATTTTCACATGGGACGAAGTAAAAGGTAAGACATCCATGTGTGTCGGCAAGAGAAACAAAGGCATAAAACTATAGGAGAAATTATTATGGCTATGACAAAGAAATTTAAAGTGTCCTTCGACGTTACCGCAAAGATGTCGTCTGACGTTCAGGCAATCTTAGAGAAAGATATGCTGCATCTATGTAAGCAGGTCGGCTCAGGTGCGATTGTCCCCAATGGTAAACAGAAGGAAATGATTGTCCAGTTCCTGACACACGGTATGGAAGGATTGATGACATTCGTAGTACGTACATCATTTCGTGAGGCCATTAAGGACATGCACGAAGAGTATGCAGATAAGGACTCTTTCAAACAATCTCCTGCAACAGTACGGGAGGTGTTCTGA‑TGTCTGACTACCTGAAAGTGCTGCAAGCAATCAAAAGTTGCCCTAAGACTTTCCAGTCCAACTATGTACGGAACAATGCGAGCCTCGTAGCGGAGGCCGCTTCCCGTGGTCACATCTCG  >  NC_001604.1/16670‑17269`

  

Alignment Legend

Aligned base mismatch/match (shaded by quality score): `ATCG/ATCG` `< 3 ≤` `ATCG/ATCG` `< 10 ≤` `ATCG/ATCG` `< 22 ≤` `ATCG/ATCG` `< 34 ≤` `ATCG/ATCG` `< 38 ≤` `ATCG/ATCG`

Unaligned base: `atcg`    Masked matching base: `atcg`    Alignment gap: `‑`    Deleted base: `‑`

Reads not counted as support for junction

read\_name Not counted due to insufficient overlap past the breakpoint.

read\_name Not counted due to not crossing MOB target site duplication.
